# Small RNA and Transcriptome Sequencing Reveal a Potential miRNA-Mediated Interaction Network That Functions during Somatic Embryogenesis in *Lilium pumilum* DC. Fisch.

**DOI:** 10.3389/fpls.2017.00566

**Published:** 2017-04-20

**Authors:** Jing Zhang, Bingyang Xue, Meizhu Gai, Shengli Song, Nana Jia, Hongmei Sun

**Affiliations:** Key Laboratory of Protected Horticulture of Education Ministry and Liaoning Province, College of Horticulture, Shenyang Agricultural UniversityShenyang, China

**Keywords:** somatic embryogenesis, miRNA expression profiling, miRNA target genes, high-throughput sequencing, *Lilium*

## Abstract

Plant somatic embryos are widely used in the fields of germplasm conservation, breeding for genetic engineering and artificial seed production. MicroRNAs (miRNAs) play pivotal roles in somatic embryogenesis (SE) regulation. However, their regulatory roles during various stages of SE remain unclear. In this study, six types of embryogenic samples of *Lilium pumilum* DC. Fisch., including organogenic callus, embryogenic callus induced for 4 weeks, embryogenic callus induced for 6 weeks, globular embryos, torpedo embryos and cotyledon embryos, were prepared for small RNA sequencing. The results revealed a total of 2,378,760 small RNA reads, among which the most common size was 24 nt. Four hundred and fifty-two known miRNAs, belonging to more than 86 families, 57 novel miRNAs and 40 miRNA^*^s were identified. The 86 known miRNA families were sorted according to an alignment with their homologs across 24 land plants into the following four categories: 23 highly conserved, 4 moderately conserved, 15 less conserved and 44 species-specific miRNAs. Differentially expressed known miRNAs were identified during various stages of SE. Subsequently, the expression levels of 12 differentially expressed miRNAs and 4 targets were validated using qRT-PCR. In addition, six samples were mixed in equal amounts for transcript sequencing, and the sequencing data were used as transcripts for miRNA target prediction. A total of 66,422 unigenes with an average length of 800 bp were assembled from 56,258,974 raw reads. Gene Ontology (GO) and Kyoto Encyclopedia of Genes and Genomes (KEGG) enrichment indicated that 38,004 and 15,497 unigenes were successfully assigned to GO terms and KEGG pathways, respectively. Among the unigenes, 2,182 transcripts were predicted to be targets for 396 known miRNAs. The potential targets of the identified miRNAs were mostly classified into the following GO terms: cell, binding and metabolic process. Enriched KEGG analysis demonstrated that carbohydrate metabolism was the predominant pathway in *Lilium* SE. Thus, we performed systemic characterization, homology comparisons and profiling of miRNA expression, and we constructed an miRNA-target network during *Lilium* SE for the first time. Our findings establish a foundation for the further exploration of critical genes and elucidation of SE in *Lilium*.

## Introduction

microRNAs (miRNAs) are a class of endogenous, small non-coding RNAs of approximately 19–24 nt in length that originate from the 3′- or 5′-arm of hairpin-folded, single-stranded RNAs. Mature miRNAs and the AGO protein combine to form the RNA-induced silencing complex (RISC), which can recognize target genes according to the complementary nature of base pairs and mediate target gene cleavage or translation repression depending on the degree of complementarity. Many advanced and effective methods have been widely used for miRNA identification, such as microarray hybridization, northern hybridization and *in situ* hybridization (ISH). In the past 5–6 years in particular, the increasing number of sequenced small RNAs has promoted research investigating novel miRNAs (Kozomara and Griffiths-Jones, 2014). Data from miRBase (v21, 2014) show a total of 28,645 hairpin precursor miRNAs and 35,828 mature miRNA products in 223 species. In the plant kingdom, miRNAs have been identified in 53 dicotyledonous and 12 monocotyledonous plants, and for most of these plants, information for the entire genome has been generated; examples of these taxa include the dicotyledonous plants *Arabidopsis thaliana* (325 precursors, 427 mature), *Carica papaya* (79 precursors, 81 mature), *Gossypium hirsutum* (78 precursors, 80 mature) and *Solanum tuberosum* (224 precursors, 343 mature) and the monocotyledonous plants *Brachypodium distachyon* (317 precursors, 525 mature), *Oryza sativa* (592 precursors, 713 mature), and *Zea mays* (172 precursors, 321 mature). Based on reports of miRNA identification in different species, the functions of miRNAs have become more apparent. Accumulating data suggest that miRNAs play crucial roles in development (Jia et al., 2014; Liu et al., 2014; Li T. et al., 2015; Wang et al., 2015; Xie et al., 2015; Bai et al., 2016; Zhang H. et al., 2016), hormone signal transduction (Qiao et al., 2012; Wójcik and Gaj, 2016), lipid metabolism (Ye et al., 2014), secondary metabolite synthesis (Li F. et al., 2015) and responses to stress (Feng et al., 2015; Candar-Cakir et al., 2016).

Embryogenesis is central to the life cycle of more than 80% of green plants (Xiang et al., 2011). Somatic embryogenesis (SE) is defined as the transition of somatic cells into somatic embryos *in vitro* (Zimmerman, 1993) and spans two phases: restructuring from somatic cells to embryogenic cells and regeneration of somatic embryos from embryogenic cells (Yang and Zhang, 2010). The developmental stages of SE are similar to zygotic embryogenesis. Both processes involve bipolar development of the shoot apical meristem (SAM)/root apical meristem (RAM), an independent tracheal system separated from the maternal tissue, which is frequently of single-cell origin (Omar et al., 2016). Because of these similarities, SE is not only the ultimate reflection of cell totipotency but also an excellent model system for studying the early stages of embryogenesis (Zimmerman, 1993; Quiroz-Figueroa et al., 2006; Nowak et al., 2015). Currently, SE is used for seedling propagation (Yang et al., 2013; Nakhooda and Mandiri, 2016), germplasm conservation (Bakhshaie et al., 2010; Pullman et al., 2016), artificial seed production (Micheli and Standard, 2016) and breeding for genetic engineering (Corredoira et al., 2016).

SE has been reported in *Arabidopsis thaliana* (Gaj, 2001), *Phoenix dactylifera* L. (Naik and Al-Khayri, 2016), *Theobroma cacao* L. (Ajijah et al., 2016), *Gladiolus hybridus* (Wu J. et al., 2015), and *Cassia occidentalis* L. (Naz et al., 2015). However, the understanding of the mechanism underlying the formation of somatic embryos is still in its infancy, and SE remains the least understood pattern of regeneration (Fehér, 2015). Subsequent to the decoding of the interaction between miRNAs and AGO, miRNAs have become accepted as master regulators through the targeting of genes involved in SE. Recently, research investigating miRNAs that regulate SE has resulted in different levels of progress in our understanding of this topic in *Arabidopsis thaliana* (Nodine and Bartel, 2010), *Oryza sativa* (Luo et al., 2006), hybrid yellow poplar (*L. tulipifera* × *L. chinense*) (Li et al., 2012), *Larix leptolepis* (Zhang et al., 2010; Li et al., 2013; Li W. F. et al., 2014), *Gossypium spp*. (Yang et al., 2013), *Dimocarpus longan* Lour. (Lin and Lai, 2013; Lin et al., 2015a,b) and *Citrus sinensis* (Wu et al., 2011; Wu X. M. et al., 2015). The same miRNA may participate in different stages of SE in different plants. For example, miR156, a highly conserved miRNA family, plays a major role in embryogenic callus differentiation in maize (Shen et al., 2013) and is involved in cotyledon embryo (CE) development in larch (Zhang et al., 2012) and longan (Lin and Lai, 2013); however, it is necessary for globular embryo (GE) development in cotton (Yang et al., 2013). Alternatively, some miRNAs play roles during the same stage of SE in different species. For example, miR167 can regulate CE development by targeting *ARF* (auxin regulatory factor) in citrus (Wu et al., 2011), cotton (Yang et al., 2013), larch (Zhang et al., 2012), and longan (Lin et al., 2015a). Furthermore, the same miRNA may regulate SE by targeting different genes. During SE in larch, miR398 participates in pre-embryo proliferation by targeting *Cu/Zn superoxide dismutase* genes (Zhang et al., 2012), whereas in longan, it is involved in cotyledon-embryo formation by targeting *DlCSD2a* (Lin and Lai, 2013). In addition, multiple miRNAs collectively regulate a specific stage of SE. As reported in longan, approximately 72% of miRNAs are differentially expressed during CE development, such as miR156, miR160, miR162, miR166, miR168, and miR390 (Zhang et al., 2012). In summary, different miRNAs may mediate SE regulation among a variety of plants, and the membership, function and target genes of the miRNAs may present distinct specificity.

*Lilium* spp. are well-established, attractive horticulture plants with ornamental value as well as considerable economic value, characterized by high market demand. As a modern vegetative propagation technique, *in vitro* regeneration via somatic embryos seems to be a promising and effective technique for the bulb cultivation and genetic improvement of these species. However, somatic embryos have been reported in few lilies species, such as *Lilium ledebourii* (Bakhshaie et al., 2010), *Lilium longiflorum* (Ho and Lai, 2006; Nhut et al., 2006), and *Lilium martagon* (Kedra and Bach, 2005). Previous studies on this topic focused mainly on plant growth regulator (PGR) supplementation and explant selection, without focusing on the molecular mechanism of SE. The available research examining *in vitro* regeneration in *Lilium* using somatic embryos is insufficient to resolve the challenges related to seed production and genetic transformation in this genus. Therefore, there is a need to elucidate the mechanism responsible for SE in *Lilium* and to thus provide valuable information for the artificial regulation and application of SE in this genus. To this end, embryogenic cultures at different stages of SE in *Lilium pumilum* DC. Fisch. were used separately to sequence small RNAs. We identified sequence information for miRNAs and detected differentially expressed miRNAs during SE in *Lilium* using computational analysis and qRT-PCR validation. Furthermore, based on the transcriptome sequencing data, we performed prediction, annotation and Gene Ontology/Kyoto Encyclopedia of Genes and Genomes (GO/KEGG) enrichment analyses of potential miRNA targets. These findings complete our knowledge of miRNAs and allow for the preliminary construction of the miRNA-target network of SE in *Lilium*, laying the foundation for the further identification of critical genes and the elucidation of the mechanism underlying SE in *Lilium*.

## Materials and methods

### Plant materials and total RNA extraction

As described previously (Zhang J. et al., 2016), there are two major stages of somatic embryo regeneration in *Lilium pumilum* DC. Fisch.: embryogenic callus induction and somatic embryo formation. In the present study, embryogenic callus induced in MS medium supplemented with a combination of picloram and α-naphthaleneacetic acid (NAA) was harvested at 4 weeks (EC1) and 6 weeks (EC2) to represent different stages of embryogenic callus induction, and GEs, torpedo embryos (TEs), and CEs were collected during somatic embryo formation based on morphological features. Non-embryogenic callus (i.e., organogenic callus, NEC) was obtained using MS medium containing a combination of 6-benzyladenine (BA) and NAA via organogenesis of *Lilium pumilum* DC. Fisch. (Zhang J. et al., 2016). Each sample represented a pool of more than 10 independent individuals. A total of six uniform growth samples (NEC, EC1, EC2, GEs, TEs, and CEs) was stored at −80°C until total RNA extraction.

Total RNA was extracted from each sample using CTAB buffer, as described in our previous report (Li et al., 2011). However, for RNA enrichment, LiCl was replaced with isopropanol to avoid the loss of small RNAs. RNA integrity was evaluated using 1% (w/v) agarose gels (Invitrogen, CA, USA). The RNA concentration and A260/280 ratio, as well as the A260/230 absorbance ratio, were determined using an Infinite® 200 PRO (Tecan, Männedorf, Switzerland). Only RNA samples with qualified A260/280 values between 1.9 and 2.1 and A260/230 values >2.0 were used for further analysis.

### cDNA library construction and transcriptome sequencing

The cDNA library was constructed using the TruSeq RNA sample prep kit (Illumina, San Diego, CA, USA) according to the manufacturer's instructions. Initially, total RNA was extracted from mixed samples of NEC, EC1, EC2, GEs, TEs, and CEs with equal weights. Subsequently, mRNA was purified from total RNA using oligo (dT)-conjugated magnetic beads. First-strand cDNA was then synthesized via the cleavage of short mRNA fragments. After the synthesis of second-strand cDNA using DNA polymerase I and RNase H, the cDNA fragments were subjected to end repair, the addition of a single “A” base and adapter ligation. Finally, the purified products were enriched through PCR to generate cDNA libraries using the HiSeq 2000 platform at Shanghai Personal Biotechnology Cp., Ltd. (Shanghai, China). Raw Illumina sequences were deposited in the National Center for Biotechnology Information Databank (NCBI) (accession number: SRP102354).

### Small RNA library construction and small RNA sequencing

Small RNA libraries were constructed using the Illumina TruSeq small RNA sample prep kit (Illumina, San Diego, CA, USA) according to the manufacturer's instructions. Briefly, 10 μg of total RNA from six independent samples was ligated to a 3′ adapter and a 5′ adapter using T4 RNA ligase. The resulting samples were reverse-transcribed using Superscript II reverse transcriptase. After amplification, the final PCR products were sequenced on the HiSeq 2000 platform at Shanghai Personal Biotechnology Cp., Ltd. (Shanghai, China). The small RNA dataset was available in the NCBI (accession number: SRP102357).

### Assembly and gene functional annotation of the transcriptome sequences

After filtering, the raw sequencing reads were used for further analysis. Sequences containing the adaptor sequence, bases with a quality score < Q20 using a 5-bp 3′ to 5′ window, reads with a final length < 25 bp, and reads with uncertain bases were removed. *De novo* transcriptome assembly of filtered raw reads into contigs and transcripts was performed using Trinity (http://trinityrnaseq.sourceforge.net/).

All transcripts were searched against the NCBI Nr (non-redundant) database (http://www.ncbi.nlm.nih.gov/) and the Swiss-Prot database (http://www.gpmaw.com/html/swiss-prot.html) using the BLAST program with an *e* <10^−5^. The transcripts with the top hits were selected as unigenes. Open reading frames (ORFs) were predicted using the GetORF program contained in the EMBOSS software package. The Blast2GO program was used for GO annotation (http://www.geneontology.org), and the unigenes were aligned to the eggNOG (evolutionary genealogy of genes: non-supervised orthologous groups) database (http://www.ncbi.nlm.nih.gov/COG/) to identify functional categories. The KEGG database (http://www.genome.jp/kegg/) was used for pathway annotation. All searches were conducted using an *e*-value cut-off of 10^−5^.

### Bioinformatics analysis of small RNA sequences

The raw reads generated from small RNA libraries were filtered to obtain clean reads by removing the 3′ and 5′ adaptors, sequences shorter than 18 nt, and low-quality reads. Sequences without repetitive sequences and with a length ranging from 15 to 30 nt were selected as unique sequences. These unique sequences were then mapped to the *Lilium* mRNA transcriptome sequences derived as described in Section Assembly and Gene Functional Annotation of the Transcriptome Sequences. To detect known non-coding RNAs (rRNAs, tRNAs, snRNAs, and snoRNAs), unique sequences were searched against the Rfam (11.0) database (http://www.sanger.ac.uk/Software/Rfam) using the BLAST tool with no mismatches. Additionally, the unique sequences were compared to identify putative known miRNAs in miRBase v21 (http://www.mirbase.org/index.shtml) using the BLAST program, and only reads with no more than two mismatches with the available miRNAs were considered known miRNAs in *Lilium*. Novel miRNAs in *Lilium* were predicted from unannotated small RNAs using Mireap software (http://sourceforge.net/projects/mireap/), and their secondary structures were explored using the Mfold program based on criteria described by Meyers et al. (2008). The expression of the miRNAs was normalized to reads per million (RPM), as reported by Murakami et al. (2006), and the expression of an miRNA family was calculated as the sum of the known miRNAs in that family (Xie et al., 2015). Statistical analysis of miRNA expression was conducted based on the fold change, which was calculated using the following formula: fold-change = log_2_ (sample1/sample2), as reported by Marsit et al. (2006). We defined a significant change in the expression of the miRNAs and miRNA families as a fold-change ≥2 or ≤ −2. Finally, the potential target genes of the identified miRNAs were predicted using the Web-based psRNATarget program (http://plantgrn.noble.org/psRNATarget/). The transcript database derived as described in Section Assembly and Gene Functional Annotation of the Transcriptome Sequences was used as a custom target database according to the criteria described previously (Allen et al., 2005).

### Validation of differentially expressed miRNAs using qRT-PCR

To validate the expression levels and roles of miRNAs during SE in *Lilium pumilum* DC. Fisch., we chose 12 differentially expressed miRNAs and four target genes for expression analysis through qRT-PCR using an ABI 7500 Real-Time PCR System (Life Technologies, CA, USA). The stem-loop primers for miRNAs were designed as reported by Varkonyi-Gasic et al. (2007). The miRNA forward primers were designed based on the miRNA sequences, but the reverse primer was universal (Table S1). Primers for targets were designed using Primer Premier 5.0 software. Total RNA was extracted from six samples, as described in 2.1, and 1 μg of total RNA from each sample was reverse transcribed into cDNA using M-MLV reverse transcriptase (Promega, Madison, USA). The 20-μL PCR mixture consisted of 2 μL of 10 × diluted template, 0.2 μM of each primer combination and 10 μL of 2 × UltraSYBR Mixture (CWBIO, Beijing, China). The amplification program for miRNAs was as follows: 95°C for 10 min, followed by 40 cycles of 95°C for 30 s and 60°C for 30 s. qRT-PCR for target genes was performed as described by Li X. Y. et al. (2014). The reactions were incubated in 96-well PCR plates (Corning, NY, USA), and each experiment consisted of three biological and technical replicates. The relative miRNA and target gene expression levels were calculated using the 2^−ΔΔCt^ method with normalization to lpu-miR159a and the *F-box family protein* (*FP*), respectively, as endogenous controls (Zhang et al., 2017).

## Results

### Transcriptome sequencing and assembly

RNA sequencing of the mixture of six samples produced a total of 56,258,974 raw reads, including 93.15% Q20 bases with a 53.79% GC content. After filtration, 85.47% of the raw reads, totaling 48,085,283 clean reads, were selected for further analysis. Using Trinity, these clean reads were assembled into 66,422 unigenes with an average length of 800 bp and a maximum length of 11,266 bp. The length of the N50 contig was 1,173 bp, with 14,013 unigenes exhibiting longer sequences. The size distribution of the unigenes indicated that the majority ranged from 200 to 500 bp, which accounted for 46.14% of the unigenes. Unigenes ranging from 500 to 1,000 bp and from 1,000 to 2,000 bp in length accounted for approximately 27.39 and 20.00% of the unigenes, respectively, while unigenes longer than 2,000 bp accounted for 6.47%.

### Gene annotation and functional classification in transcriptome sequencing

After searching the eggNOG database, we classified the unigenes into 25 categories (Figure S1). Among these, the top category was function unknown, which contained 13,862 (21.49%) unigenes, followed by general functional prediction only (18.01%), post-translational modification, protein turnover, and chaperones (8.29%), signal transduction mechanisms (8.08%), transcription (5.96%) and carbohydrate transport and metabolism (4.41%); cell motility was the least common functional group, accounting for only approximately 0.01% of the unigenes.

A total of 38,004 unigenes were annotated using Blast2GO. The greatest distribution of biological processes (Table S2) corresponded to the metabolic process category (31.40%), followed by cellular process (26.59%), response to stress (5.70%), and multicellular organismal development (3.70%). Additionally, there were 780 and 2,153 unigenes involved in embryo development and post-embryonic development, respectively. In the cellular component category (Table S2), many of the unigenes were clustered in cell (28,527), intracellular (27,178) and cytoplasm (22,509) categories. With respect to molecular function, the three main representative distributions were binding (25,632), receptor activity (202) and structural molecule activity (1,303). A large proportion of the unigenes was classified into the binding category (approximately 94.45%). Finally, we summarized the 41 GO functional terms related to embryogenesis (Table S3). Most of the GO terms were distributed among embryo development (213 unigenes), terminating in seed dormancy (447 unigenes), root development (275 unigenes), embryo sac development (156 unigenes) or seed germination (110 unigenes).

All unigenes were subjected to KEGG enrichment to identify the biological functions of the *Lilium* unigenes. The 15,497 unigenes mapped to the KEGG database (Table S4) were classified into 6 main categories, including 341 pathways. Metabolism was the largest category, accounting for 36.68% of the unigenes, followed by human disease, genetic information processing, organismal systems, environmental information processing and cellular processes, which accounted for 19.38, 17.56, 11.61, 7.19, and 7.58% of the unigenes, respectively. It is noteworthy that the largest proportion of the metabolism category was carbohydrate metabolism (7.58%), indicating this type of metabolism was active during *Lilium* SE. Annotation of the unigenes using the KEGG database will facilitate further dissection of the specific biological processes and pathways that occur in *Lilium* and lay the foundation for elucidating the role of new genes in non-model plants.

### Overview of small RNA sequencing data

**Figure 1:**
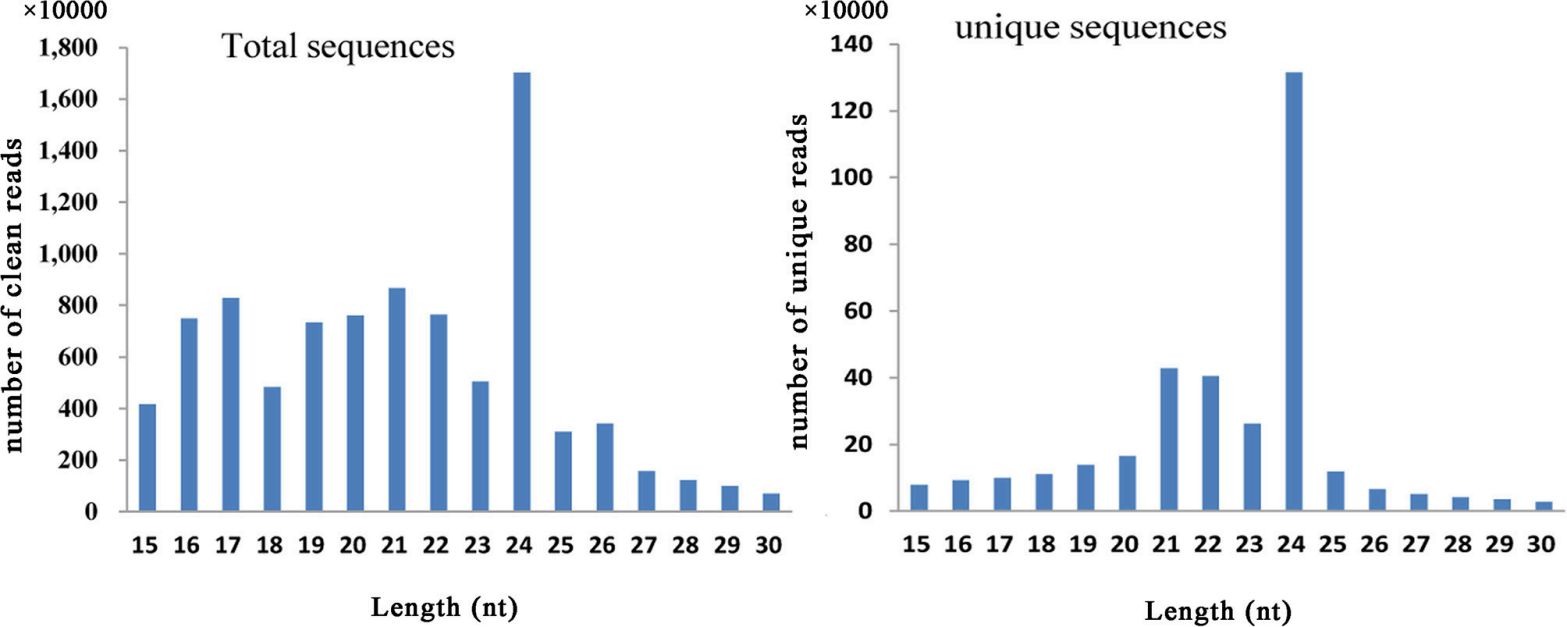
**Distribution of small RNAs among different categories in ***Lilium pumilum*** DC. Fisch**.

In this study, six small RNA libraries from different embryogenic cultures were constructed and sequenced. These samples included three different types of callus (NEC, EC1, and EC2) collected during callus induction, as well as samples collected from three time points (GE, TE, and CE) during somatic embryo formation. After removing low-quality reads, a total of 14,468,038–23,162,793 raw reads and 11,855,198–19,010,244 clean reads were obtained for each library (Table S5). The length distribution of the small RNAs (15–30 nt) revealed that a length of 24 nt was the most abundant class among both clean and unique reads (Figure 1). Reads with a length of 21 nt accounted for the second largest distribution.

The unique sequences were further annotated into different RNA classes against the Rfam database using BLAST. A total of 2,378,760 sRNAs were annotated in six libraries, and the highest abundance (465,522) was observed in CE (Table 1). Among non-coding RNAs (rRNA, tRNA and snoRNA), rRNA represented the main distribution, accounting for 37 and 43% of total sRNA in the unique and total sequences, respectively (Table 1). Additionally, the unique sequences were employed to align the miRNA homologs using miRBase v21. In the six small RNA libraries, a total of 147,386 unigenes were aligned with known miRNAs. The most frequent distribution was observed for CE (28,875), which accounted for 6.20% of sRNA (465,522) (Table 1).

**Table 1:** **Summary of Rfam annotation**.

**Samples**	**Unique sequences**						
	**NECs**	**EC1**	**EC2**	**GEs**	**TEs**	**CEs**	**Total**
tRNA	30,748	22,226	24,096	31,824	28,151	33,867	170,912
rRNA	143,751	132,894	142,823	162,179	139,026	167,614	888,287
snoRNA	36,051	31,454	27,249	37,643	34,611	42,843	209,851
miRNA	25,959	21,067	20,317	26,965	24,203	28,875	147,386
intro	13,692	11,235	12,709	14,536	12,794	15,618	80,584
others	148,918	119,472	122,096	166,295	148,254	176,705	881,740
total	399,119	338,348	349,290	439,442	387,039	465,522	2,378,760

### Identification of known miRNAs in *Lilium*

**Figure 2:**
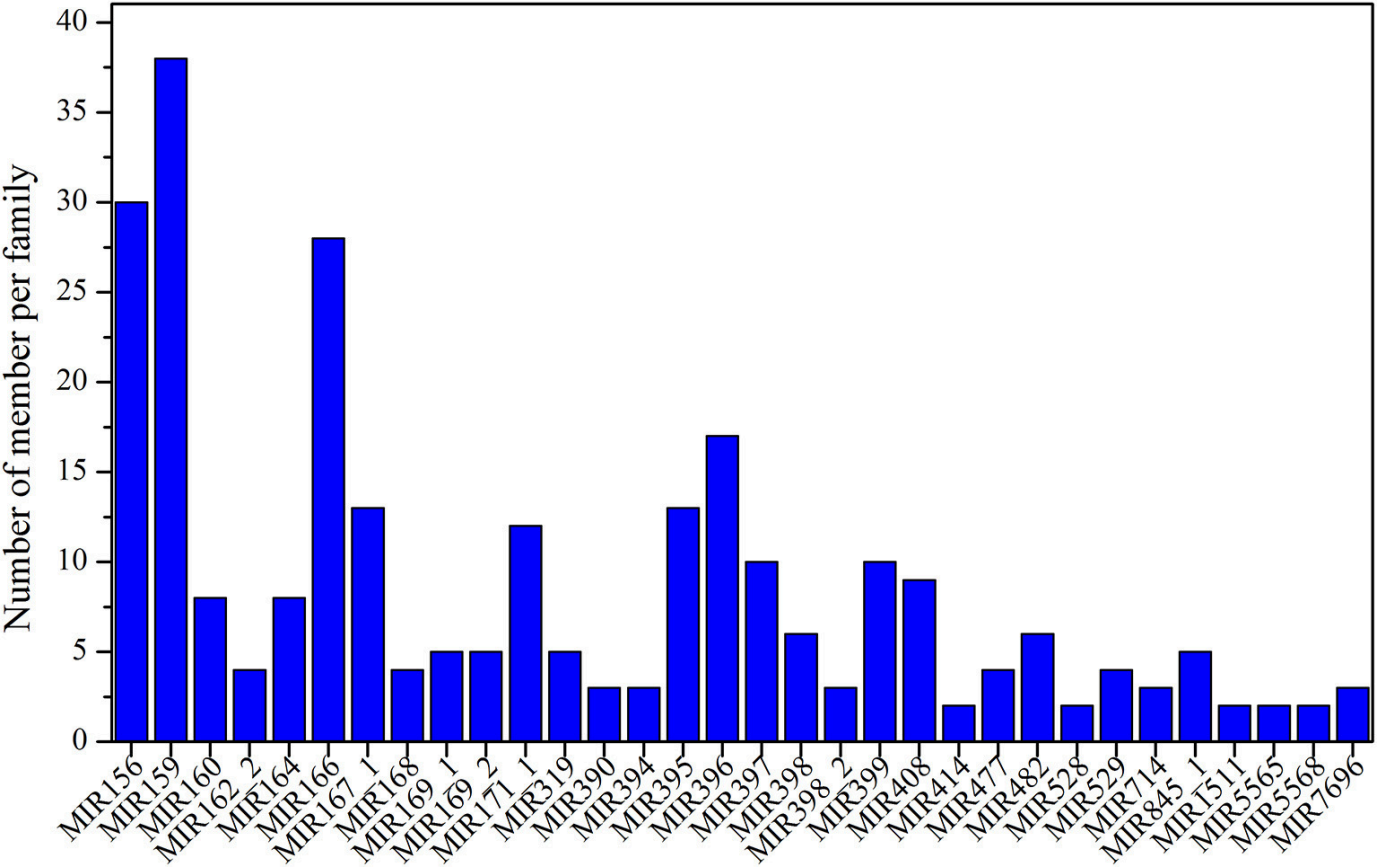
**Distribution of known miRNA family sizes in ***Lilium pumilum*** DC. Fisch**.

After analyzing the results of the alignment against miRbase v21, a total of 452 known miRNAs were identified in the six samples. The numbers of identified miRNAs are shown in Table S6; the largest number was observed for NEC (327) and the smallest for EC2 (270). The identified known miRNAs belong to more than 86 miRNA families, and information regarding the miRNA family members, mature sequences and lengths is detailed in Table S7. Among these 86 miRNA families, more than two-thirds consisted of only one member, such as miR164_2, miR827, miR1510, and miR2118 (Table S7). Some miRNA families included multiple members (Table S7, Figure 2), such as miR160, miR390, miR408 and miR529, among which miR159 was the largest family, comprising 38 members, followed by miR156 (30), miR166 (28), miR396 (17), miR167_1 (13), and miR395 (13).

### Evolutionary roles of known miRNAs in *Lilium*

**Figure 3:**
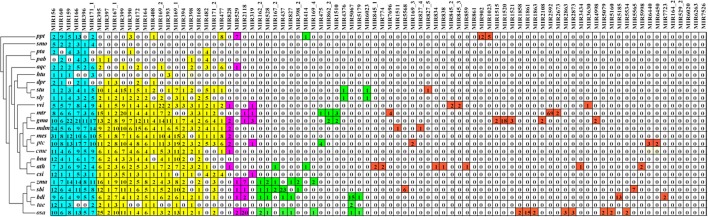
**Comparison of 86 known miRNA families in ***Lilium pumilum*** DC. Fisch. and their homologs in 24 land plants**. Known miRNA families of *Lilium* identified from small RNA sequencing are listed in the top row. The number in each well corresponds to the related miRNA family size obtained from miRBase v21. The colors represent relative miRNA families classified into different groups with similar conservation. Blue, yellow, purple, green and red represent relative miRNA families with homologs across more than 20, 10-19, 5-9, 2-4, and 1 plant species, respectively. *Ppt, Physcomitrella patens*; *smo, Selaginella moellendorffii*; *pta, Pinus taeda*; *pab, Picea abies*; *aqc, Aquilegia caerulea*; *htu, Helianthus tuberosus*; *dpr, Digitalis purpurea*; *stu, Solanum tuberosum*; *sly, Solanum lycopersicum*; *vvi, Vitis vinifera*; *mtr, Medicago truncatula*; *gma, Glycine max*; *mdm, Malus domestica; mes, Manihot esculenta*; *cme, Cucumis melo*; *ptc, Populus trichocarpa*; *bna, Brassica napus*; *ath, Arabidopsis thaliana*; *csi, Citrus sinensis*; *zma, Zea mays*; *sbi, Sorghum bicolor*; *bdi, Brachypodium distachyon*; *osa, Oryza sativa*; *tae, Triticum aestivum*.

To investigate the evolutionary roles of the known miRNAs identified in *Lilium*, these miRNAs were subjected to additional comparisons against 24 other land plants, including five monocotyledons (*Zea mays, Sorghum bicolor, Brachypodium distachyon, Oryza sativa, Triticum aestivum*), 15 dicotyledons (*Arabidopsis thaliana, Vitis vinifera, Glycine max, Aquilegia caerulea, Helianthus tuberosus, Digitalis purpurea, Solanum tuberosum, Solanum lycopersicum, Medicago truncatula, Malus domestica, Manihot esculenta, Cucumis melo, Populus trichocarpa, Brassica napus, Citrus sinensis*), two gymnosperms (*Pinus taeda, Picea abies*), one bryophyte (*Physcomitrella patens*) and one lycophyte (*Selaginella moellendorffii*). As shown in Figure 3, the 86 known miRNA families exhibited different numbers of homologous sequences in the 24 compared species. Here, we separated these miRNA families of *Lilium* into four categories according to the BLAST results. The first group consisted of highly conserved miRNA families with homologous sequences in more than 10 plants. There were 23 such miRNA families: miR156, miR160, miR159, miR166, miR396, miR171_1, miR395, miR397, miR167_1, miR399, miR390, miR172, miR164, miR408, miR169_2, miR393, miR169_1, miR394, miR398, miR168, miR482, miR171_2, and miR477. It was remarkable that six miRNA families were conserved across more than 20 species (miRNA families shaded in blue). Second, some miRNA families were conserved across 5–9 plant species, corresponding to the moderate level. This category consisted of four members: miR828, miR529, miR2118, and miR319. A total of 15 miRNA families made up the third group. These miRNAs were conserved in 2–4 species and were classified as showing a low level of conservation, including miR162_2, miR528, miR167_2, miR437, miR827, miR398_2, miR414, miR169_4, miR475, miR862_2, miR1510, miR4376, miR5067, miR5179, and miR6023. Finally, the fourth group contained species-specific miRNAs representing 44 miRNA families. These miRNAs were found in only one species and included miR845_1, miR774, miR7696, miR1511, miR5568, miR169_3, miR827_4, miR164_2, miR529_2, miR6020, miR6263, and miR7526.

### Analysis of the expression abundance of known miRNA families in *Lilium*

**Figure 4:**
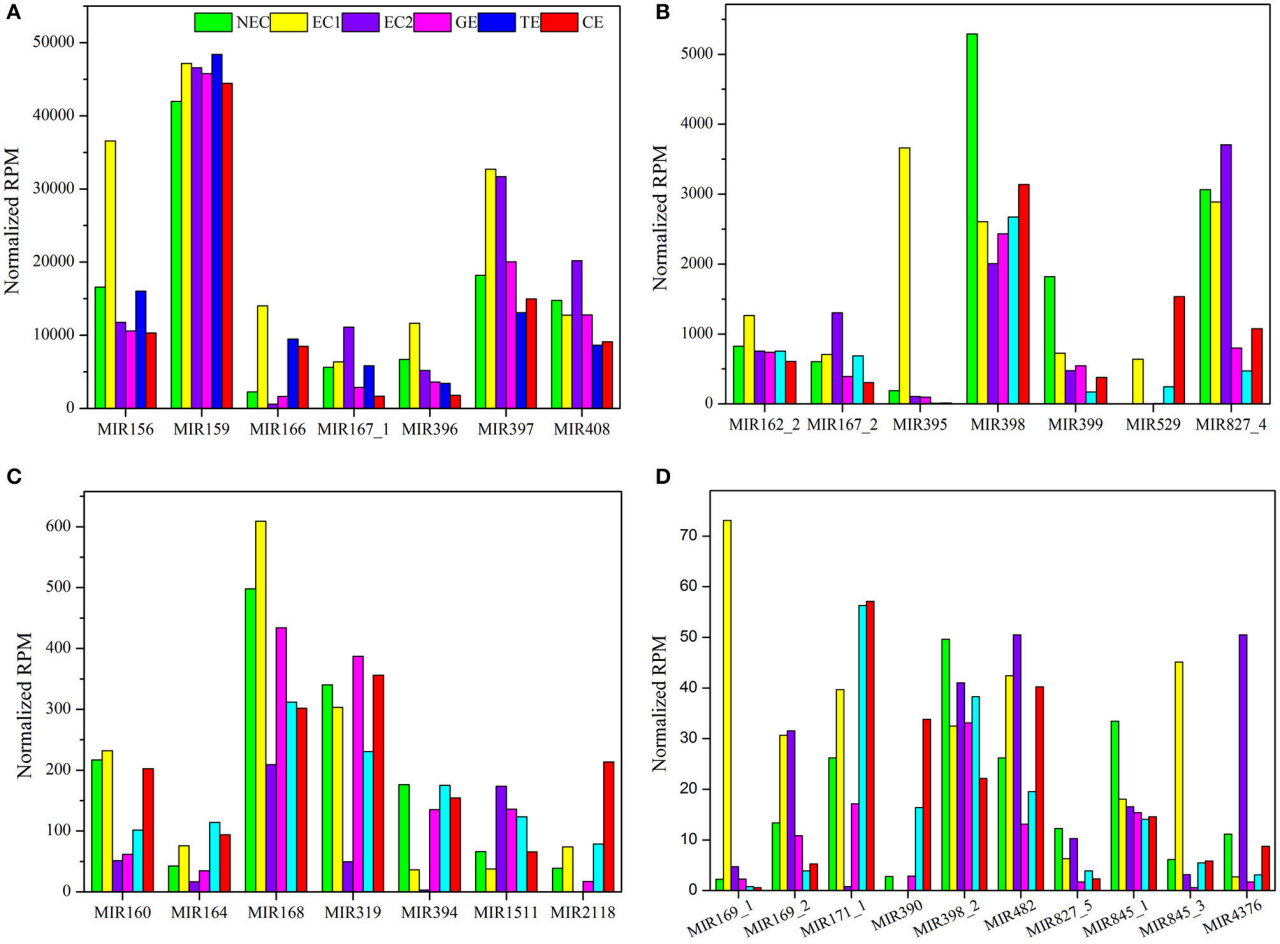
**Reads per million known miRNAs in ***Lilium pumilum*** DC. Fisch. (A)** miRNA families with highest expression abundance over 10,000 RPM; **(B)** miRNA families with highest expression abundance between 1,000 and 10,000 RPM; **(C)** miRNA families with highest expression abundance between 100 and 1,000 RPM; **(D)** miRNA families with highest expression abundance between 10 and 100 RPM.

The sequencing data indicated that the expression of the 86 known miRNA families differed markedly, ranging from fewer than 10 to more than 45,000 RPM among the six libraries (Table S8). These 86 known miRNA families were classified into five groups based on their maximum expression abundance. Among the 86 miRNA families, seven miRNA families (miR156, miR159, miR166, miR167_1, miR396, miR397, and miR408) expressed more than 10,000 RPM in at least one sample, and miR159 was the most abundant miRNA family, with its expression levels exceeding 40,000 RPM in all samples (Figure 4A), followed by seven miRNA families (miR162_2, miR167_2, miR395, miR398, miR399, miR529, and miR827_4). The maximum expression levels ranged from 1,000 to 10,000 RPM (Figure 4B). miR395 exhibited the highest expression level in EC1, far exceeding the levels in the other five samples. The third group contained seven miRNA families (miR160, miR164, miR168, miR319, miR394, miR1511, and miR2118), with expression levels ranging from 100 to 1,000 RPM. In this group, miR168 and miR319 displayed higher expression levels than did the other five miRNA families (Figure 4C). There were 10 miRNA families in the fourth group with expression levels ranging from 10 to 100 RPM: miR169_1, miR169_2, miR171_1, miR390, miR398_2, miR482, miR827_5, miR845_1, miR845_3, and miR4376 (Figure 4D). Finally, the remaining 55 miRNA families were all expressed at levels lower than 10 RPM, as shown in Table S8.

### Analysis of the expression profiles of known miRNAs during SE in *Lilium*

**Figure 5:**
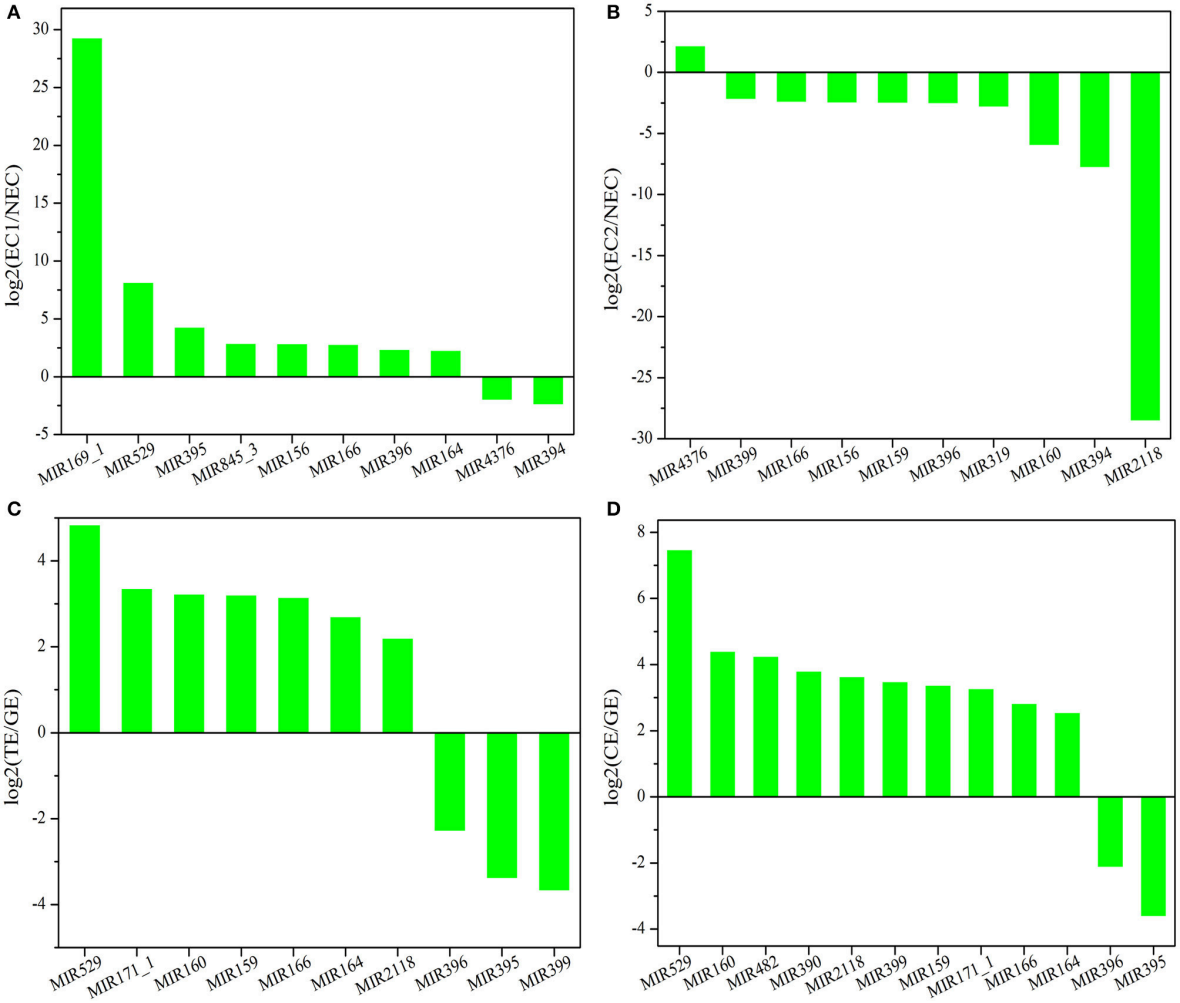
**Differentially expressed known miRNA families in ***Lilium pumilum*** DC. Fisch. (A)** comparison of differentially expressed known miRNA families between NEC and EC1; **(B)** comparison of differentially expressed known miRNA families between NEC and EC2; **(C)** comparison of differentially expressed known miRNA families between GE and TE; **(D)** comparison of differentially expressed known miRNA families between GE and CE.

**Figure 6:**
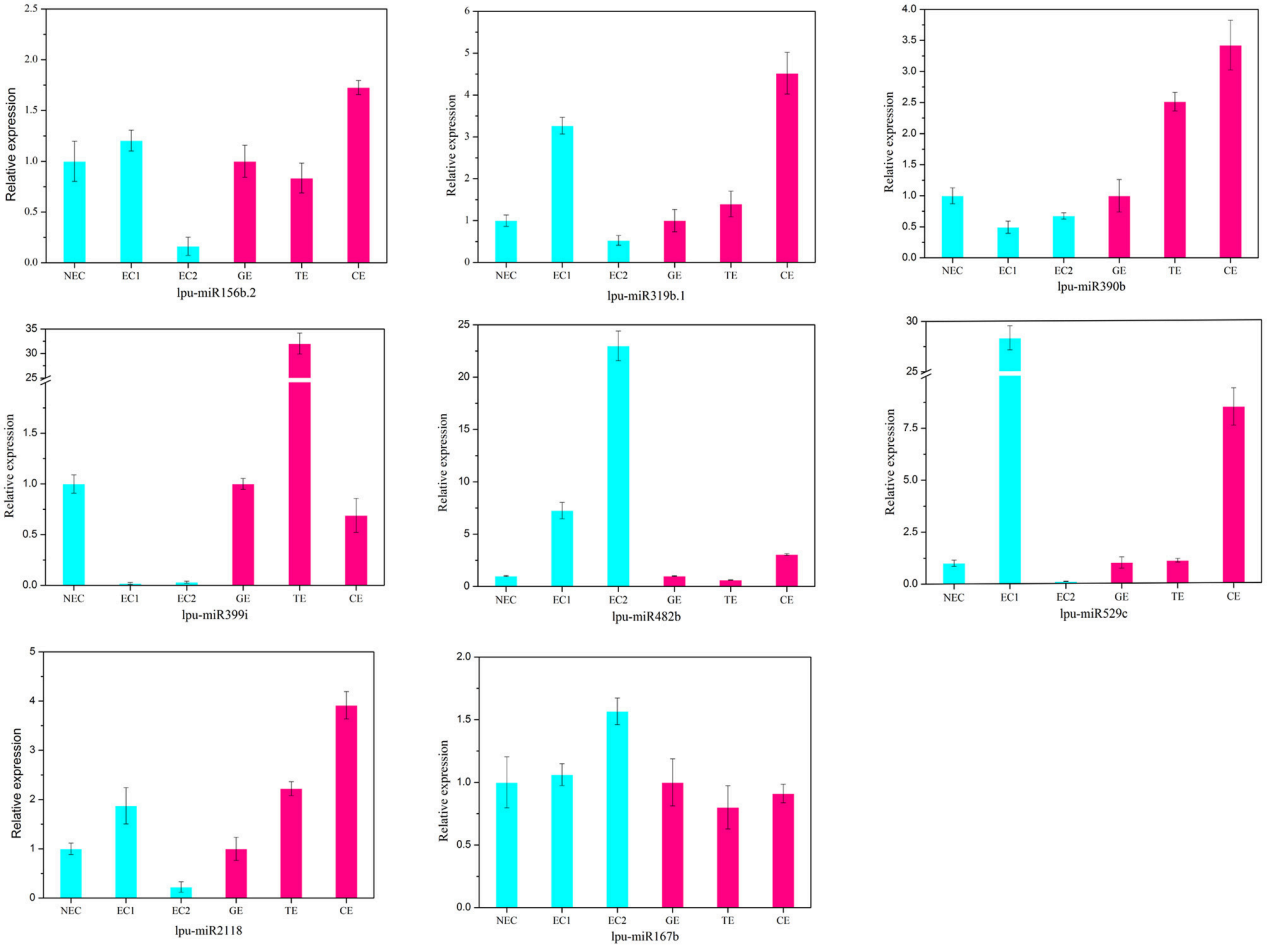
**Validation and comparison of miRNAs with different expression levels using qRT-PCR**. NEC, non-embryogenic callus; EC1, embryogenic callus induced in 4 weeks; EC2, embryogenic callus induced in 6 weeks; GE, globular embryo; TE, torpedo embryo; CE, cotyledon embryo. The qRT-PCR values are the means ± SE of three replicates.

**Figure 7:**
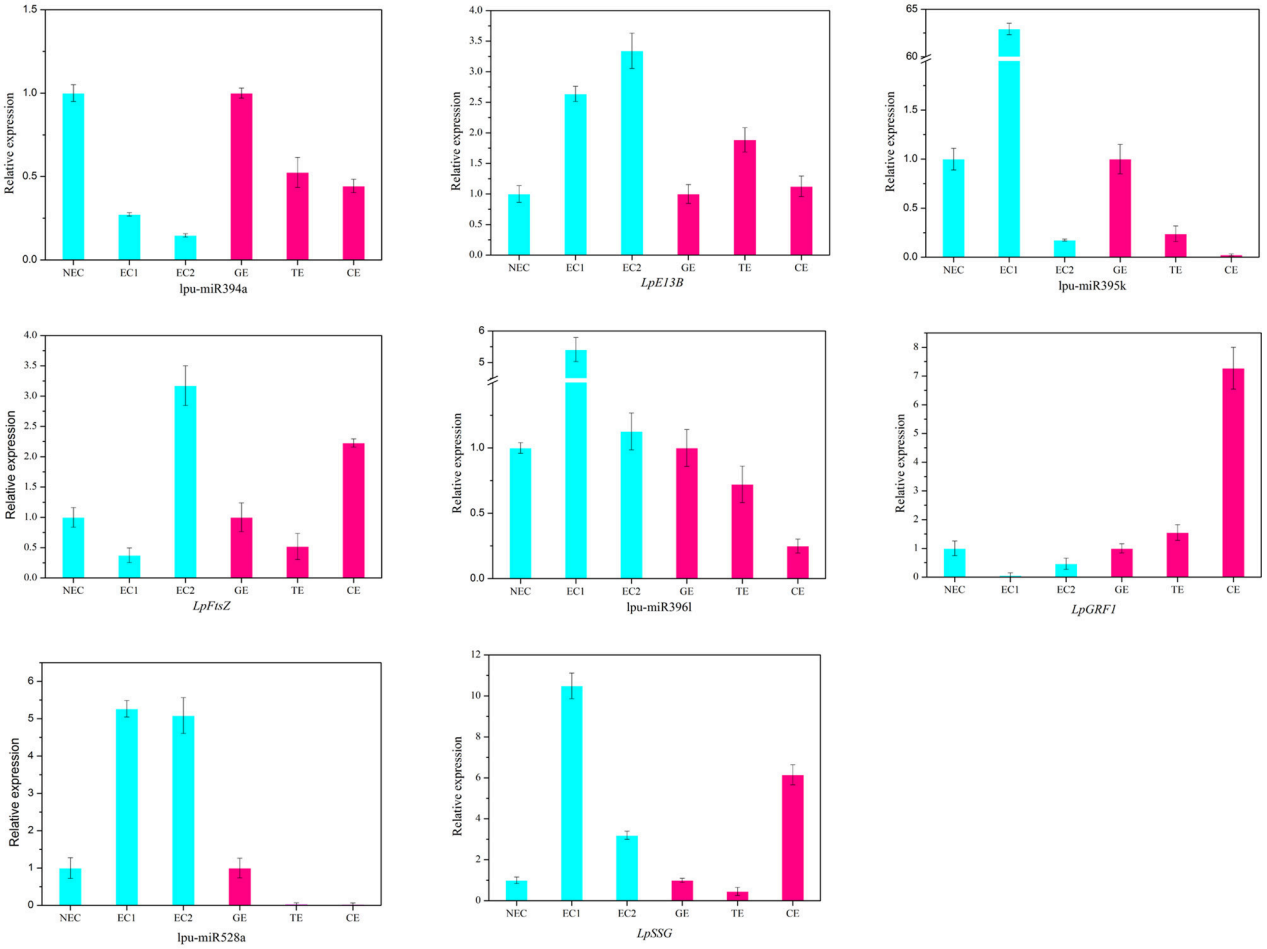
**Expression profiles of 4 miRNAs and 4 targets during ***Lilium*** SE**. *LpE13B* (c44501_g1_i1) targeted by lpu-miR394a; *LpFtsZ* (c46557_g1_i1) targeted by lpu-miR395k; *LpGRF1* (c36759_g1_i2) targeted by lpu-miR396l; *LpSSG* (c44221_g1_i1) targeted by lpu-miR528a. NEC, non-embryogenic callus; EC1, embryogenic callus induced in 4 weeks; EC2, embryogenic callus induced in 6 weeks; GE, globular embryo; TE, torpedo embryo; CE, cotyledon embryo. The qRT-PCR values are the means ± SE of three replicates.

There are two major stages of *Lilium* SE: embryogenic callus induction and somatic embryo formation. NEC and GEs were considered as controls for both stages. miRNA families exhibiting an absolute fold-change value ≥ 2 were deemed to be differentially expressed. During embryogenic callus induction, 10 differentially expressed miRNA families were identified in EC1 and EC2 in comparison with NEC (Figures 5A,B). A comparison between EC1 and NEC revealed 8 miRNA families with varying degrees of up-regulated expression, excluding miR4376 and miR394, which were down-regulated (Figure 5A). However, the majority of the differentially expressed miRNA families were down-regulated in EC2 compared with NEC, and miR2118 presented the greatest fold-change. Regarding somatic embryo formation, 10 and 12 differentially expressed miRNA families were identified in TEs and CEs, respectively, compared with GEs (Figures 5C,D). Among the 10 differentially expressed miRNA families compared between TEs and GEs, 7 up-regulated and 3 down-regulated miRNA families were identified. Peak fold-changes were observed for up-regulated miR529 and down-regulated miR399, followed by up-regulated miR171 and miR160 and down-regulated miR395 and miR396 (Figure 5C). Similarly, up-regulated miR529 and down-regulated miR395 and miR396 were identified compared with CE-GE (Figure 5D). Among these differentially expressed miRNAs, the expression levels of 11 miRNAs with remarkably different expression patterns and 1 miRNA with a steady expression profile were further investigated via qRT-PCR. The expression trends of the other detected miRNAs coincided with the data obtained from small RNA sequencing (Table S8, Figures 6, 7). As shown in Figures 6, 7, miR394a, miR399i, and miR390b were down-regulated in EC, and their expression levels peaked in GEs, TEs and CEs, respectively, during somatic embryo formation. In addition, the expression of miR319b.1, miR529c, miR482b, and miR2118 was up-regulated in EC1 and EC2, and marked accumulation occurred in CEs during somatic embryo formation (GEs, TEs, and CEs). While the expression of miR395k, miR396l, and miR528a was up-regulated in EC1, it decreased continuously during somatic embryo formation. To further validate the miRNA-mediated mRNA silencing during SE in *Lilium*, the expression of four predicted targets was tested. Some negative correlation between miRNAs and target mRNAs are presented in Figure 7. The diverse expression profiles of the miRNAs indicated that different miRNA families might play different regulatory roles during various stages of SE, suggesting that a complicated miRNA-mediated regulation network might be involved in SE, which would allow a broader understanding of the regulatory role of miRNAs in *Lilium* SE.

### Identification of potential novel miRNAs in *Lilium*

In addition to known miRNAs, non-annotated small RNA sequences were employed to predict novel *Lilium* miRNAs using Mireap software. Novel miRNA candidates with the same mature sequence but originating from different loci were considered to belong to a novel miRNA family. Herein, a total of 57 novel miRNA families representing 61 unique sequences were identified (Table S9). These novel miRNA candidates were given names in the form of “lpu-miRn plus number” (e.g., lpu-miRn1), using a, b, and c to differentiate members from the same novel miRNA family. With the exception of lpu-miRn2, lpu-miRn3 and lpu-miRn8, the other novel miRNA families contained only one member. The majority of these novel miRNAs had a length of 21 nt (75.4%, Table S9). Novel miRNA precursors with lengths ranging from 39 to 93 nt and a minimum free energy (MFE) ranging from −18.3 to −55.1 kcal/mol for secondary hairpin structures were identified. Among the novel miRNAs, 40 miRNA^*^s exhibited an expression level that was much lower than that of the corresponding miRNAs. Thirty-four miRNA^*^s presented more than 1 read in six libraries, excluding lpu-miRn14^*^, lpu-miRn18^*^, lpu-miRn26^*^, lpu-miRn27^*^, lpu-miRn48^*^, and lpu-miRn51^*^, further supporting the presence of these novel miRNAs (Table S9).

### Prediction of potential targets of known miRNAs

To provide biological insight into the miRNA-mediated pathways that play a role in *Lilium* SE, a total of 66,422 sequences from the mRNA transcriptome data obtained herein were used as a custom target database for known miRNAs because no completely sequenced genome of *Lilium* is available. The results revealed 389 known miRNAs (86.06%) that aligned with 2,156 potential target sequences (Table S10). Among the miRNA-target pairs, more than 80% of the miRNAs putatively targeted multiple unigenes (ranging from 2 to 164), while 44 miRNAs exhibited a single unigene. Conversely, <15% of the unigenes were targeted by more than 2 miRNAs up to a total of 22 miRNAs. These miRNA-target pairs support the existence of complicated roles of these miRNAs during *Lilium* SE.

As shown in Table S10, the predicted target genes were involved in a broad variety of biological processes and included transcription factors (TFs) and protein-coding genes related to development, hormones and resistance; carbohydrate metabolism-related enzymes; and signal transduction-related receptor kinases. For example, the TFs containing *cup-shaped cotyledon 1* (*CUC1*), *squamosa promoter-binding-like protein* (*SPL*), and *scarecrow-like protein 6* (*SCL6*) were identified as target genes of miR156, miR529 and miR171, respectively. The response of some target genes to PGRs was also detected in *Lilium* SE. The *auxin response factor* (*ARF*) family comprises a class of TFs that typically function at the core of the auxin transcription signaling pathway by regulating the expression of auxin response genes. Here, we identified 6 members of the *AFR* family: *ARF2*, targeted by miR4414; *ARF10*, −*17*, and −*18*, targeted by miR160; *ARF12*, targeted by miR167; and *ARF21*, targeted by miR7698. miR393 was also found to participate in the auxin signal transduction pathway by regulating the *transport inhibitor response 1-like* (*TIR1*) protein. In addition to the TFs related to development and hormones, miR164, miR169 and miR319 were indicated to be involved in resistance to stress by regulating the *NAC domain-containing protein* (*NAC*), *nuclear transcription factor Y* (*NF-Y*) and *proliferating cell factor 5* (*PCF5*), respectively. Moreover, some receptor kinases were identified as target genes of miRNAs. miR390 and miR399 were predicted to target *serine/threonine-protein kinase* (*PBS1*) and *somatic embryo receptor kinase* (*SERK*), suggesting that both miRNAs are involved in transcription signaling. In addition, *starch synthase 3* (*SS3*) and *beta-fructofuranosidase 4* (*INV4*), targeted by miR159; *granule-bound starch synthase 1* (*SSG1*), targeted by miR528; *fructose-1,6-bisphosphatase* (*F16P2*), targeted by miR5658, miR394 and miR395; and *plastidial pyruvate kinase 4* (*PKP4*), targeted by miR845 are critical enzymes involved in the metabolism of starch, sucrose and glycolysis, suggesting that these miRNA-target pairs are related to carbohydrate metabolism.

**Figure 8:**
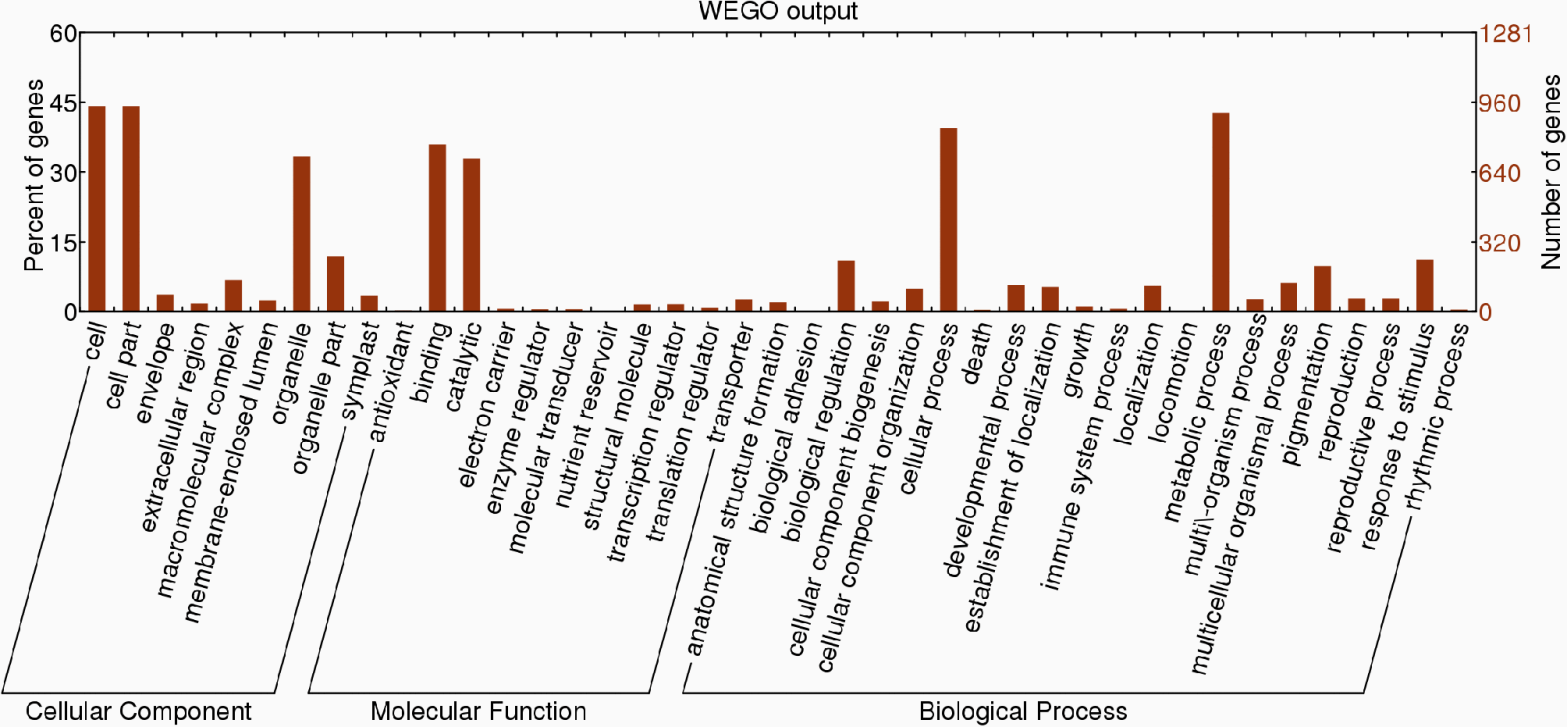
**Functional classification of targets of known miRNAs according to Gene Ontology (GO) categories**. Enriched GO data were plotted using WEGO (http://wego.genomics.org.cn/cgi-bin/wego/index.pl). The left Y-axis represents the percentages of targets of known miRNAs in each main category. The right Y-axis indicates the numbers of targets of known miRNAs in each GO category.

GO category analysis of all targets of known miRNAs was performed to obtain annotations of miRNA functions during *Lilium* SE. Among the 2,156 targets of 389 known miRNAs, 1,281 targets exhibited GO terms (Table S11). Among the cellular component categories, cell, cell part and organelle comprised the largest proportion of targets. Most of the targets were categorized into the binding and catalytic molecular function categories, while the majority of targets in the biological process categories were classified into cellular and metabolic process (Figure 8). Furthermore, the enriched GO terms for the targets of differentially expressed known miRNAs were analyzed in four groups (NEC-EC1, NEC-EC2, GEs-TEs, and GEs-CEs; Figure S1). As shown in Figure S2, the distribution of enriched GO terms in the four groups was similar to that shown in Figure 8, with the exception of nucleoside binding, nucleotide binding, transferase and cellular process during embryogenic callus induction (Figure S2a) and nucleoside binding, nucleotide binding and multicellular organismal process during somatic embryo formation (Figure S2b). miRNAs with targets that played a role in biological processes related to SE are summarized in Table S12. miR529 might play a role in embryo development ending in seed dormancy, embryonic pattern specification and embryonic meristem initiation, whereas miR399 might be related to embryonic development and post-embryonic development. Some miRNAs were also related to organ development, such as miR156 and miR160, which regulate leaf morphogenesis, and miR169, which regulates root morphogenesis. Moreover, a portion of the miRNAs may participate in hormone metabolism. miR169, miR166 and miR160 were related to the auxin-activated signaling pathway; miR395 and miR529 were related to the gibberellic acid-mediated signaling pathway and regulation of the gibberellic acid-mediated signaling pathway; and miR164 may participate in the response to jasmonic acid.

In addition, an enriched KEGG analysis was performed for the targets of the differentially expressed miRNAs (Figures S3–S6). During embryogenic callus induction, the predominant KEGG pathway was carbohydrate metabolism, followed by energy metabolism and lipid metabolism (Figures S3, S4). However, carbohydrate metabolism, lipid metabolism and nucleotide metabolism were the significantly enriched pathways during somatic embryo formation (Figures S5, S6).

## Discussion

### Identification and conservation analysis of miRNAs in *Lilium*

Plant miRNAs were first identified in *Arabidopsis* in 2002 (Reinhart et al., 2002). With the development of high-throughput technology and further research in plant genome sequencing, the identification and functional annotation of plant miRNAs have advanced dramatically over the past 10 years. Numerous miRNAs have been discovered in different plant species, such as *Lycopersicon esculentum* (Candar-Cakir et al., 2016), *Camellia sinensis* (Song H. et al., 2016), *Musa* spp. (Ghag et al., 2015), *Brassica napus* (Machado et al., 2015), *Populus tomentosa* (Chen et al., 2016), *Glycine max* (Wang Y. et al., 2016), and *Vitis vinifera* L. (Kullan et al., 2015). However, information regarding miRNA sequences has been published in the miRNA database for only a few ornamental flowers, including *Aquilegia caerulea, Digitalis purpurea, Helianthus annuus* and *Helianthus tuberosus*. *Lilium* spp. are abundant bulbous flowers worldwide, but their genome and miRNAs have not yet been explored. Information on key genes and miRNAs can be obtained through deep sequencing (Candar-Cakir et al., 2016; Cheng et al., 2016; Zhang W. et al., 2016; Zou et al., 2016). Thus, using this method, we pioneered research on the identification of miRNAs during SE in *Lilium*. Compared with the 253 known miRNAs from 61 miRNA families, 102 known miRNAs from 23 miRNA families and 83 known miRNAs from 35 miRNA families identified in yellow poplar (Li et al., 2012), maize (Shen et al., 2013) and larch (Zhang et al., 2012), respectively, *Lilium* exhibits more diverse miRNAs and miRNA families. Furthermore, the prediction of miRNA-target pairs revealed greater possibilities compared with the results reported for *Lycium barbarum* L. (Zeng et al., 2015) and *Lycopersicon esculentum* (Candar-Cakir et al., 2016). These findings suggest that a more complex gene network is mediated by diverse miRNAs during SE in *Lilium*.

miRNAs have been discovered in almost all eukaryotes, and plant miRNAs present highly conserved sequences (Rajagopalan et al., 2006) and highly similar functions (Ambros, 2004; Zhang et al., 2007) among different plant species. Within the group of highly conserved miRNAs in *Lilium*, miR156, miR159, miR160, and miR166 exhibited sequences that were homologous to the common ancestor of almost all embryophytes (Zhang et al., 2006; Xie and Zhang, 2015); miR396 presented a sequence homologous to that of the common ancestor of vascular plants (Wang et al., 2015), and miR167_1, miR399 and miR172 might be related to the development of angiosperms. These miRNAs may play significant roles in a series of physiological and biotechnological processes. The moderately conserved miR828 has only been found in rosids, indicating that it might have originated from a common rosid ancestor. Among the less conserved miRNAs, miR862_2 and miR1510, as well as miR4376 and miR6023, have only been observed in Fabales and Solanales, respectively. In addition, approximately half of the *Lilium* miRNAs are species-specific miRNAs. For example, miR902 and miR1023 exhibit a single homolog only in bryophytes (*Physcomitrella patens*), suggesting that some of the miRNAs identified in *Lilium* are ancient. These species-specific miRNAs are novel in evolutionary history and arose under particular conditions in specialized plants, usually to produce new functions, but with much lower expression levels compared with those of highly conserved miRNAs (Ma et al., 2010; Tang, 2010).

### Potential regulatory networks of miRNAs during *Lilium* SE

**Figure 9:**
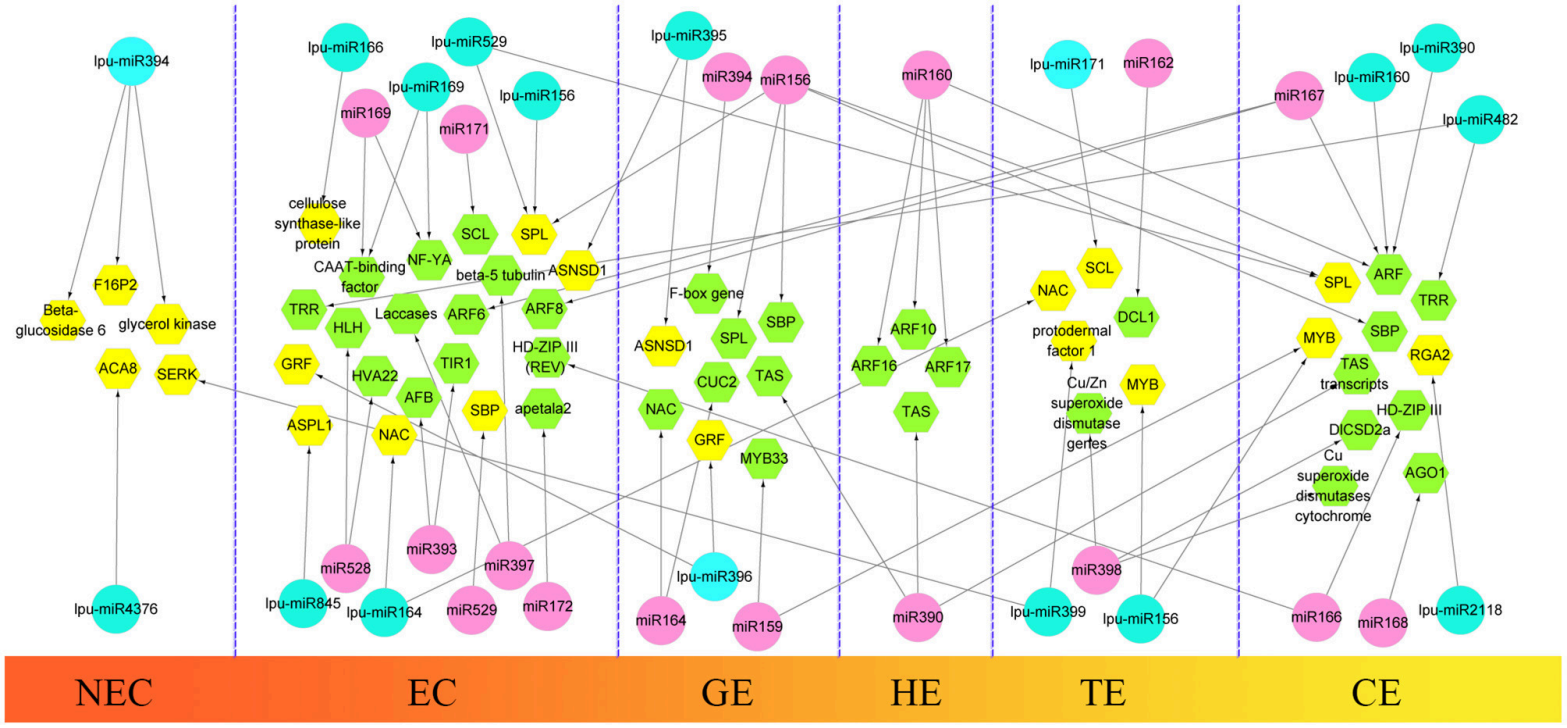
**Comparison of hypothetical schematic model of miRNA-mediated interaction network during SE in ***Lilium pumilum*** DC. Fisch. and other plants**. The miRNAs and potential targets in *Lilium pumilum* DC. Fisch. are shaded in blue and yellow, respectively. The black arrows denote the relationship between miRNAs and potential targets identified and predicted in this study. The miRNAs and potential targets in other plants are shaded in pink and green, respectively. The black arrows denote the relationship between miRNAs and potential targets according to previous reports listed in Table S13.

During the first step of SE, somatic cells acquire embryogenic potential and transform into a mass of embryogenic cells. This step is defined as the induction of the embryogenic callus. In this study, we selected 3 types of callus in *Lilium pumilum* DC. Fisch., including NEC, EC1, and EC2 (forming a GE in subculture) to construct 3 small RNA libraries to obtain differentially expressed miRNAs between non-embryogenic and embryogenic callus and to explore critical miRNAs during embryogenic callus induction in *Lilium*. During the next step of SE, the embryogenic callus differentiated into somatic embryos through three successive forms—GE, TE and CE. These samples of *Lilium pumilum* DC. Fisch. were evaluated through small RNA sequencing. Differentially expressed miRNAs were also selected during somatic embryo formation in *Lilium*. In addition to evaluating the expression levels of the miRNAs, target prediction was performed to annotate the function of the miRNAs. The results were then compared with the miRNA-target regulatory networks provided in previous reports, and potential roles for the miRNAs involved in *Lilium* SE were proposed (Figure 9).

The highly conserved miR156 family is involved in leaf patterning (Xie et al., 2012; Ghag et al., 2015), floral morphogenesis (Wang et al., 2009), juvenile-adult transitions (Wu and Poethig, 2006) and fiber elongation (Xie et al., 2015) through regulation of *SPL* expression. Moreover, miR156 plays a critical role in SE. As reported for *Arabidopsis thaliana*, miR156 presented significant up-regulation at the eight-cell stage, resulting in the suppression of the accumulation of *SPL10* and *SPL11* (Nodine and Bartel, 2010). Similarly, as the largest family with the most abundant miRNA expression, miR156 participates in early SE in yellow poplar by targeting *SPL3/5/10/11* (Li et al., 2012). In longan and larch, miR156 targets *SPL* but is also involved in the formation of CE (Lin and Lai, 2013; Ye et al., 2014). In the present study, the expression profile of miR156 is presented in Figure 6, supporting its involvement in CE by targeting *SPL2/11/13/19* (Table S10). The inverse trend in the EC2 expression (Figure 6) may benefit somatic embryo formation (Wu et al., 2011) because callus in the EC2 stage already possesses embryogenic potential.

The miR529 sequence exhibits relatively high similarity to that of miR156, sharing 14–16 nt (Cuperus et al., 2011). In eudicot species lacking miR529, miR156 may compensate for this loss and facilitates the diversification of eudicot *SPL* (Morea et al., 2016). During embryogenic callus induction in maize, miR529 accumulates dramatically and potentially contributes to the dedifferentiation of immature embryos (Shen et al., 2013). In *Lilium*, the expression level of miR529 (Figure 6) suggests its involvement in initial embryogenic callus induction and CE development.

miR166 has been implicated in the regulation of CEs during SE in citrus and larch by targeting *HD-ZIP III* (Wu et al., 2011; Zhang et al., 2012). In *Lilium*, a diverse expression profile of miR166 was detected: it accumulated in EC1, TEs, and CEs but was expressed at lower levels in EC2 and GEs (Figure 5). These findings support a critical role of miR166 during all stages of SE in *Lilium*. This result has been confirmed in longan (Lin and Lai, 2013). As reported by Wu X. M. et al. (2015), accumulation of miR166 in the embryogenic callus can prevent embryo maturation during early SE. Unexpectedly, the previously reported target *HD-ZIP III* was not found in this study. Here, one of the predicted targets of miR166 was *cellulose synthase-like protein* (Table S10), which may promote epidermal cell separation from neighboring cells, followed by the acquisition of embryogenic potential during SE in *Eleutherococcus senticosus* (Xilin et al., 2010). Thus, miR166 may play a similar role during *Lilium* SE, and the regulation of *cellulose synthase-like protein* by miR166 during *Lilium* SE should be investigated in future analyses.

During *Lilium* SE, miR169 was the most differentially expressed miRNA family between NEC and EC1 (Figure 5A). *NF-YA*, a predicted target of miR169 (Table S10), regulates embryo development (Fornari et al., 2013; Mu et al., 2013). In larch SE, miR169 targets *NF-YA* and is down-regulated in subcultured embryogenic callus, but the function of miR169 remains unknown (Zhang et al., 2010). The present results indicate that miR169 may also participate in early SE in *Lilium* (Figure 5A).

In addition to *SPL* and *NF-YA*, some other TFs were predicted as targets in this study, such as *NAC, SCL*, and *MYB* (Table S10). *NAC* is a plant-specific TF that is targeted by miR164, participating in the regulation of meristem differentiation (Cheng et al., 2012), leaf senescence (Wu et al., 2016) and the drought response (Wang L. et al., 2016). miR164 is dramatically differentially expressed during embryogenic callus induction in maize (Shen et al., 2013). Similar results have been obtained for sweet orange (Wu X. M. et al., 2015). Here, miR164 was only down-regulated in NEC and GEs (Figure 5), which indicated that it was acquired during the initial stage of EC induction and somatic embryo formation. *MYB* is one of the largest TF families and plays a significant role in development (Zhang W. et al., 2016), flavonol and hydroxycinnamic acid biosynthesis (Liu et al., 2016), and stilbene accumulation (Wong et al., 2016). Regulation mediated by miR159 has been observed during different SE stages in different plants. miR159 plays dominant roles in GE and CE formation in sweet orange (Wu X. M. et al., 2015) and longan (Lin and Lai, 2013), respectively, and in CE formation and embryogenic callus subcultures in larch (Zhang et al., 2012; Li et al., 2013). In the present study, miR159 was up-regulated in TEs and CEs (Figures 5C,D), demonstrating a role in somatic embryo formation. As reported by Zhang et al. (2010), miR159 may impact ABA-induced signaling during SE. *SCL*, which is targeted by miR171, is a plant-specific TF that is involved in root development (Di Laurenzio et al., 1996), meristem formation (Curaba et al., 2013) and leaf morphogenesis (Guo et al., 2016). miR171 is differentially expressed during *Lilium* SE and potentially targets *SCL6* (Figure 5 and Table S10). miR171-*SCL*-mediated regulation of SE has been identified in sweet orange (Wu et al., 2011; Wu X. M. et al., 2015), radish (Zhai et al., 2014), and larch (Zhang et al., 2012).

PGRs are key factors in cell division and cell differentiation during SE (Jiménez, 2005). miRNAs and their targets involved in hormone signaling during SE have been identified in previous reports; such targets include *ARF*, which is related to auxin signaling and is targeted by miR160, miR167 and miR390. During longan SE, miR160 and miR390 are highly expressed in heart embryos (HEs) and TEs, whereas miR167 accumulates in CEs and targets *ARF3/8* (Lin and Lai, 2013; Lin et al., 2015a,b). The most abundant expression of miR390 has been observed during embryogenic callus induction in cotton, whereas miR167 regulates the expression of *ARF6/8* in GEs and CEs (Yang et al., 2013). In citrus, the formation of GEs and the transition of CEs are regulated by miR167 and miR390 (Wu et al., 2011). The development of CEs is also regulated by miR160 and miR167 (Zhang et al., 2012). During *Lilium* SE, miR160 was expressed at much lower levels in EC2 and GEs (Figures 5B,C), whereas miR390 was highly expressed in CEs (Figure 6). These findings are consistent with those reported for citrus and larch (Wu et al., 2011; Zhang et al., 2012). In addition to auxin, GAs are an important hormone during SE. As reported in larch (Zhang et al., 2010) and *Schisandra incarnata* (Sun et al., 2013), the content of GAs decreases in the later stages of SE. The changes in the GA content could affect the expression of *GRF* (Wang et al., 2014). *GRF* can impact cell proliferation, cell enlargement, and flower and leaf morphogenesis (Wu et al., 2014; Vercruyssen et al., 2015) and is targeted by miR396 (Liang et al., 2014; Li et al., 2016). In the present study, miR396l negatively regulated *GRF1* (Figure 7), indicating its requirement for early embryogenesis and advanced somatic embryo mutation by targeting *GRF*.

miR394, miR399, and miR482 are miRNAs that are differentially expressed during SE in both *Lilium* (Figure 5) and cotton (Yang et al., 2013), but they have distinct predicted targets. In cotton, *NAC, ACT7*, and *GATA-type zinc finger transcription regulators* are targeted by miR394, miR399, and miR482, respectively. However, in *Lilium*, the low expression of miR394a in EC may benefit the accumulation of *Glucan endo-1,3-beta-glucosidase* in early embryogenesis (*E13B*) (Figure 7). Therefore, we speculate that miR394 is involved in carbohydrate metabolism during SE in *Lilium*. *Leaf curling responsiveness* (*LCR*), targeted by miR394 in *Arabidopsis thaliana* (Knauer et al., 2013; Song J. B. et al., 2016), was not identified in the present study. *Protodermal factor 1* and *SERK* were targeted by miR399 (Table S10). Emergence of the protoderm has been noted as an indicator of SE (Raju et al., 2015; Zhang J. et al., 2016), and the accumulation of *SERK* is the most significant indication of the transition from somatic cells to embryogenic cells (Ma et al., 2012; Silva et al., 2014). According to our previous study examining SE in *Lilium pumilum* DC. Fisch., the protoderm appears in HE but not in TE (Zhang J. et al., 2016), which may be explained by the accumulation of miR399, which regulates *protodermal factor 1* in TE. Additionally, the accumulation of miR399 in NEC indicates that miR399 might repress the expression of *SERK* in NEC. The relationship between miR399 and both targets should be confirmed in future analyses. In addition, miR482 potentially targets *topless-related protein* (*TRR*) (Table S10), which regulates shoot/root differentiation during embryogenesis in *Arabidopsis thaliana* (Long et al., 2006). Thus, miR482 may regulate apical meristem specification of the shoot/root during *Lilium* SE.

miR528 is required for embryo dedifferentiation in maize SE, and it may regulate an *HLH transcription factor* (Shen et al., 2013). In *Lilium*, a relationship between miR528 and *HLH* was not observed. However, the predicted target -*SSG* showed a negative relationship compared with miR528a during somatic embryo mutation (Figures 6, 7), suggesting that miR528 may participate in starch metabolism during *Lilium* SE.

miR395, miR845, and miR2118 also exhibited differential expression during *Lilium* SE (Figures 7, 8), which has rarely been reported in other plants. The possible targets of miR395 and miR845 include *asparagine synthetase domain-containing protein 1* (*ASNSD1*) and *aspartic proteinase-like protein 1* (*ASPL1*), respectively (Table S10), which are involved in the synthesis of asparagine and protein processing. We infer that both miRNAs might contribute to amino acid metabolism and protein metabolism during *Lilium* SE. Additionally, *cell division protein FtsZ* (*FtsZ*) was also predicted as a target gene of miR395. The negative regulation of miR395k and *FtsZ* suggests active cell division in EC2 and CE during *Lilium* SE (Figure 6). miR2118 was found to target the disease resistance protein *RGA2* (Table S10), suggesting its involvement in the plant response to stress.

Focused on differentially expressed miRNAs, a few scientists have pioneered studies to reveal the specific function of miRNAs in SE. In the model plant *Arabidopsis thaliana*, the effects of miR167 and miR393 on SE frequency have been confirmed using transgenic lines (Su et al., 2016; Wójcik and Gaj, 2016). The most miR167 over-expressing embryogenic callus could not induce any somatic embryos by reducing the transcription of *ARF6* and *ARF8* (Su et al., 2016). Similarly, in the *MIR393* over-expression lines, SE frequency and explant sensitivity to 2,4-D were significantly reduced, suggesting the regulatory role of miR393 with respect to *TIR1* and *Auxin F-box protein 2* (*AFB2*) during embryogenic transition (Wójcik and Gaj, 2016). Thus, it is reasonable to presume that the differential expression of miRNAs may significantly affect the induction and formation of *Lilium* somatic embryos, and the expression profile of miRNAs will operate as a knocking hammer for artificial regulation in *Lilium* SE.

In conclusion, this study constitutes pioneering research focusing on the molecular mechanism of SE mediated by miRNAs using high-throughput sequencing. Based on a comprehensive analysis of the expression profile of miRNAs, prediction of targets, and GO/KEGG enrichment, we have preliminarily illustrated the regulatory network of miRNA-target-mediated SE in *Lilium*. This is the first study to provide information about miRNAs in *Lilium*, and it is a small but significant step toward the elucidation of the regulatory mechanisms that are mediated by miRNAs in plant SE.

## Author contributions

JZ and HS conceived and designed the experiments. JZ, BX, MG, and SS analyzed the high-throughput sequencing data. BX and MG contributed reagents and materials. JZ, BX, MG, and NJ performed the qRT-PCR experiment. JZ and HS wrote and revised the manuscript. All authors read and approved the final manuscript.

### Conflict of interest statement

The authors declare that the research was conducted in the absence of any commercial or financial relationships that could be construed as a potential conflict of interest.
