# Supplementary material for: Small RNA and Transcriptome Sequencing Reveal a Potential miRNA-Mediated Interaction Network That Functions during Somatic Embryogenesis in Lilium pumilum DC. Fisch
Source: Front Plant Sci. 2017 Apr 20;8:566. doi: 10.3389/fpls.2017.00566 (PMC5397531; doi:10.3389/fpls.2017.00566)
Supplement: Supplementary file 13 [file Table13.DOCX]

| **Table S13 Summary of present knowledge regarding the regulatory network of miRNAs and their targets in SE** | | | | |
| --- | --- | --- | --- | --- |
| miRNA | Targets | Function in embryogenesis | Species | Reference |
| miR156 | | | | |
|  | *SBP* | accelerate embryogenesis, and subsequently switch vegetative organ morphogenesis | rice | Luo et al. 2006 |
|  | *SBP/SCL* | regulatory function during somatic embryo induction process | Valencia sweet orange | Wu et al. 2011 |
|  | *SBP* | embryo dedifferentiation | maize | Shen et al. 2013 |
|  | *SBP* | regulatory roles during CE development | *Larix leptolepis* | Zhang et al. 2012 |
|  | *SPL* | key repression factors of transcription during the transition from the premature to maturation phase | yellow poplar | Li et al. 2012 |
|  | *SPL* | GE formation | cotton | Yang et al. 2013 |
|  | *SPL* | play regulatory roles during early stages of longan SE or CE development | longan | Lin and Lai, 2013 |
|  | *SPL* | initiate the early embryoids | citrus | Wu et al. 2015b |
|  | *SBP* | required for early SE | Arabidopsis | Nodine and Bartel, 2010 |
| miR159 | | | | |
|  | *MYB* | GE formation | Valencia sweet orange | Wu et al. 2011 |
|  |  | ABA-induced signaling during embryogenesis | *Larix leptolepis* | Zhang et al. 2010 |
|  |  | regulatory roles during CE development | *Larix leptolepis* | Zhang et al. 2012 |
|  | *MYB33* | function in middle and late stages of SE | longan | Lin and Lai, 2013 |
|  | *MYB33* | maintenance of embryogenic potential and somatic embryo maturation | *Larix kaempferi* (Lamb.) Carr. | Li et al. 2013 |
| miR160 | | | | |
|  | *ARF* | regulatory roles during CE development | *Larix leptolepis* | Zhang et al. 2012 |
|  | *ARF16/ARF17* | HE and TE formation and morphogenesis | longan | Lin and Lai, 2013 |
|  | *ARF10/ARF16/ARF17* | establish and maintain auxin signals during embryogenesis | longan | Lin et al. 2015b |
| miR162 | | | | |
|  | *DCL1* | regulatory function during total SE process, especially in single embryo to late CE | *Larix leptolepis* | Zhang et al. 2012 |
| miR164 | | | | |
|  | *NAC* | GE formation | Valencia sweet orange | Wu et al. 2011 |
|  | *CUC2* | maintaining GEs | citrus | Wu et al. 2015 |
| miR166 | | | | |
|  | *HD-ZIP* | CE embryo morphogenesis | Valencia sweet orange | Wu et al. 2011 |
|  | *HD-ZIP III* | regulatory roles during CE development | *Larix leptolepis* | Zhang et al. 2012 |
|  | *HD-ZIP III* | embryogenic cell differentiation/ development leading to GE | longan | Lin and Lai, 2013 |
|  | *HD-ZIP III (REV)* | avoid precocious activation of embryo maturation programme in EC | citrus | Wu et al. 2015 |
| miR167 | | | | |
|  | *ARF* | CE morphogenesis | Valencia sweet orange | Wu et al. 2011 |
|  | *ARF6/ ARF8* | succeeding conversion to CE | cotton | Yang et al. 2013 |
|  | *ARF* | regulatory roles during CE development | *Larix leptolepis* | Zhang et al. 2012 |
|  | *ARF3/ ARF8* | play a major role during CE and mature embryonic stages | longan | Lin and Lai, 2013 |
|  | *ARF6/ARF8* | GE formation and subsequent conversion to CE | longan | Lin et al. 2015a |
| miR168 | | | | |
|  | *AGO1* | regulatory function during somatic embryo induction process | Valencia sweet orange | Wu et al. 2011 |
|  |  | regulatory function during total SE process, in single embryo to late CE | *Larix leptolepis* | Zhang et al. 2012 |
|  |  | leading to the development of embryo | longan | Lin and Lai, 2013 |
| miR169 | | | | |
|  | *NF-Y/CCAAT-*  *binding factor* | embryo dedifferentiation | maize | Shen et al. 2013 |
|  | *NF-YA* | transformation of the embryogenic competence | *Larix leptolepis* | Zhang et al. 2010 |
| miR171 | | | | |
|  | *SLP* | regulatory function during somatic embryo induction process | Valencia sweet orange | Wu et al. 2011 |
|  | *SCL* | early cellular differentiation | *Larix leptolepis* | Zhang et al. 2010 |
|  | *SCL* | maintenance of embryogenic potential | *Larix kaempferi (Lamb.) Carr.* | Li et al. 2014 |
|  | *SCL* | exert function on PEMs/ induction process of SE | *Larix leptolepis* | Zhang et al. 2012 |
|  | *SCL* | inactivates GA pathway to promote SE | *Citrus* | Wu et al. 2015 |
| miR172 | | | | |
|  | *apetala2* | specify cell fate and adequately response  to ABA induction | *Larix leptolepis* | Zhang et al. 2010 |
| miR390 | | | | |
|  | *TAS3a* | GE embryo formation | Valencia sweet orange | Wu et al. 2011 |
|  | *TAS3 transcripts* | regulatory roles during CE development | *Larix leptolepis* | Zhang et al. 2012 |
|  | *TAS* | HE and TE formation | longan | Lin and Lai, 2013 |
| miR393 | | | | |
|  | *TIR1/AFB* | embryogenic transition induced | *Arabidopsis* |  |
| miR394 | | | | |
|  | *F-box gene* | required for GEs formation | citrus | Wu et al. 2015 |
| miR397 | | | | |
|  | *Laccases* | early morphological formation of SE | yellow poplar | Li et al. 2012 |
|  | *Laccases* | maintain embryonic cells in a thin-wall and meristematic state | rice | Luo et al. 2006 |
|  | *Laccases and beta-6 tubulin* | GE formation | Valencia sweet orange | Wu et al. 2011 |
|  | *Laccases* | regulating the thickness of the cell wall during SE | *Larix leptolepis* | Zhang et al. 2012 |
|  | *Laccases* | cell wall thickness | longan | Lin and Lai, 2013 |
| miR398 | | | | |
|  | *Copper superoxide dismutases cytochrome C oxidase subunit V* | CE morphogenesis | Valencia sweet orange | Wu et al. 2011 |
|  | *Cu/Zn superoxide dismutase genes* | modulation of PEM propagation and transition to single embryo | *Larix leptolepis* | Zhang et al. 2012 |
|  | *DlCSD2a* | promoting maturation of CE | longan | Lin and Lai, 2013 |
| miR528 | | | | |
|  | *HLH transcription regulator/ HVA22-like protein a* | embryo dedifferentiation | maize | Shen et al. 2013 |
| miR529 | | | | |
|  | *SBP* | embryo dedifferentiation | maize | Shen et al. 2013 |
