# Supplementary material for: Small RNA and Transcriptome Sequencing Reveal a Potential miRNA-Mediated Interaction Network That Functions during Somatic Embryogenesis in Lilium pumilum DC. Fisch
Source: Front Plant Sci. 2017 Apr 20;8:566. doi: 10.3389/fpls.2017.00566 (PMC5397531; doi:10.3389/fpls.2017.00566)
Supplement: Supplementary file 15 [file Image2.PDF]

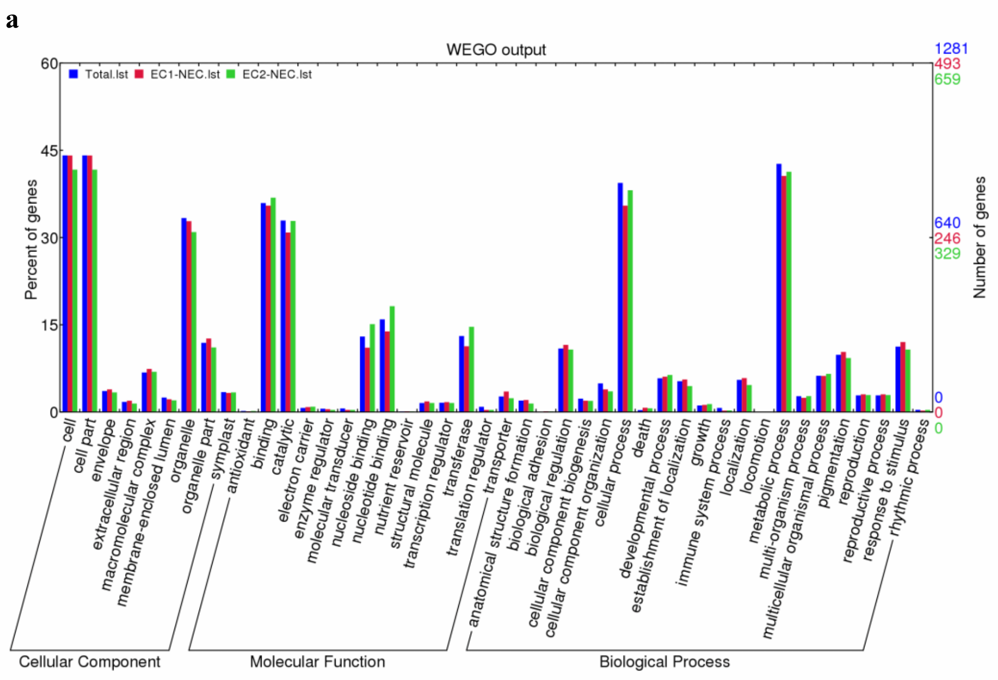

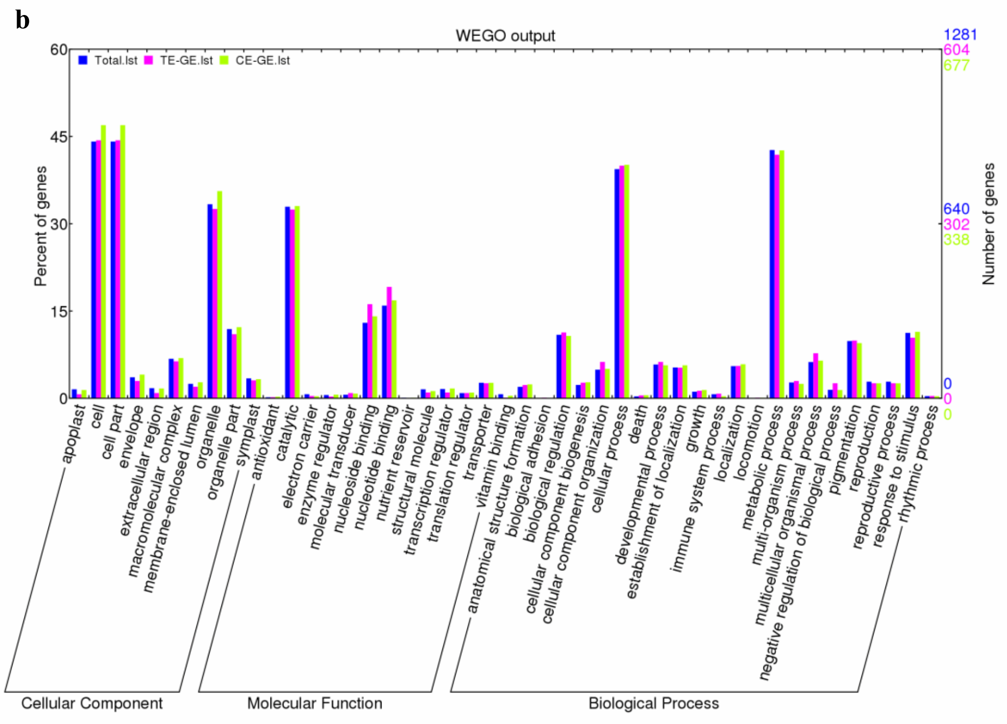

FIGURE S2 GO term enrichment analysis of differentially expressed miRNAs in *Lilium pumilum* DC. Fisch.

a: enriched GO terms during embryogenic callus induction. b: enriched GO terms during somatic embryos formation.

Note: enriched GO terms for the targets of total miRNAs were shown in total, and enriched GO terms for the targets of differentially expressed miRNAs were shown in EC1-NEC, EC2-NEC, TE-GE and CE-GE, respectively.
