# Supplementary material for: Small RNA and Transcriptome Sequencing Reveal a Potential miRNA-Mediated Interaction Network That Functions during Somatic Embryogenesis in Lilium pumilum DC. Fisch
Source: Front Plant Sci. 2017 Apr 20;8:566. doi: 10.3389/fpls.2017.00566 (PMC5397531; doi:10.3389/fpls.2017.00566)
Supplement: Supplementary file 17 [file Image4.PDF]

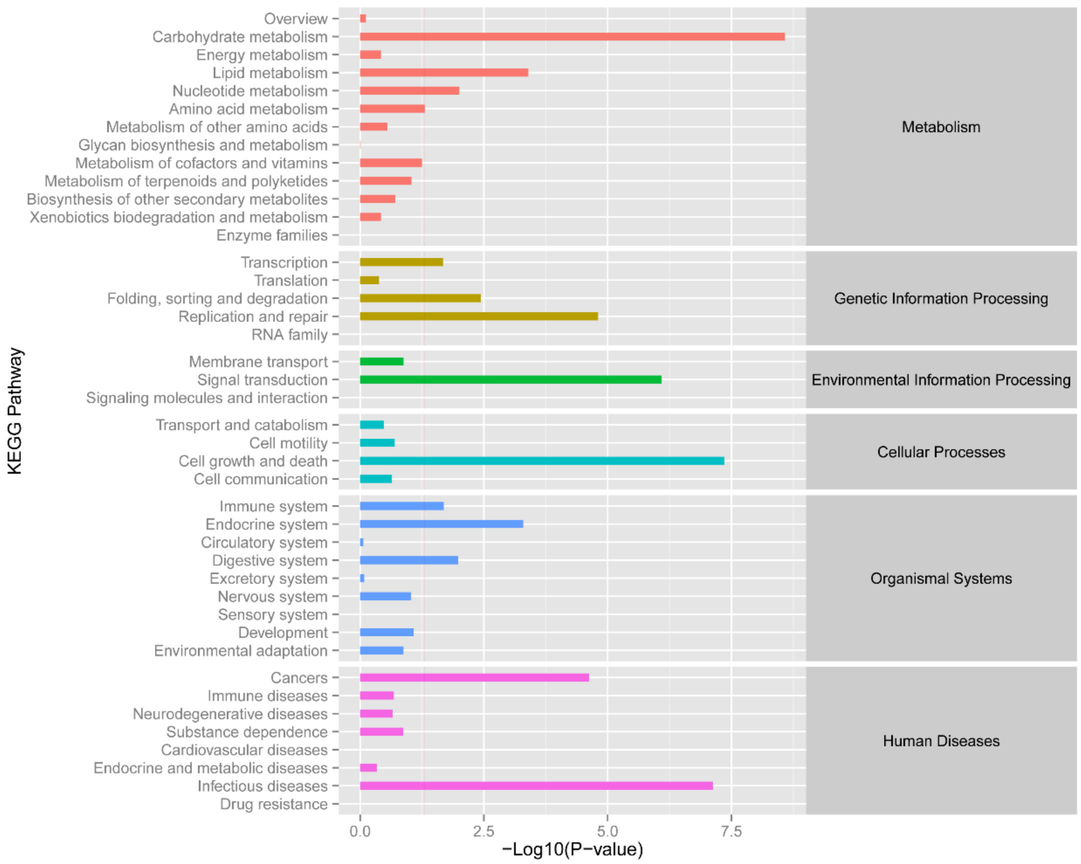

FIGURE S4 KEGG Enrichment analysis of targets of differentially expressed miRNAs between GE and TE in *Lilium pumilum* DC. Fisch.
